# Integration of Digital Phenotyping and Genomics for Dry Eye Disease: Protocol for a Prospective Cohort Study

**DOI:** 10.2196/67862

**Published:** 2025-05-12

**Authors:** Ken Nagino, Yasutsugu Akasaki, Nobuo Fuse, Soichi Ogishima, Atsushi Shimizu, Akira Uruno, Yoichi Sutoh, Yayoi Otsuka-Yamasaki, Fuji Nagami, Jun Seita, Tomohiro Nakamura, Satoshi Nagaie, Makiko Taira, Tomoko Kobayashi, Ritsuko Shimizu, Atsushi Hozawa, Shinichi Kuriyama, Atsuko Eguchi, Akie Midorikawa-Inomata, Masahiro Nakamura, Akira Murakami, Shintaro Nakao, Takenori Inomata

**Affiliations:** 1 Department of Ophthalmology Juntendo University Graduate School of Medicine Tokyo Japan; 2 Department of Digital Medicine Juntendo University Graduate School of Medicine Tokyo Japan; 3 Department of Hospital Administration Juntendo University Graduate School of Medicine Tokyo Japan; 4 Department of Telemedicine and Mobile health Juntendo University Graduate School of Medicine Tokyo Japan; 5 Tohoku Medical Megabank Organization Tohoku University Sendai Japan; 6 Advanced Research Center for Innovations in Next-Generation Medicine Tohoku University Sendai Japan; 7 Division of Biomedical Information Analysis Iwate Tohoku Medical Megabank Organization, Disaster Reconstruction Center Iwate Medical University Yahaba Japan; 8 Advanced Data Science Project RIKEN Information R&D and Strategy Headquarters RIKEN Tokyo Japan; 9 Division of Biomedical Information Analysis Institute for Biomedical Sciences Iwate Medical University Yahaba Japan; 10 Department of Data Science Kyoto Women's University Kyoto Japan; 11 Department of Neurology Neuroscience & Sensory Organs Tohoku University Graduate of Medicine Sendai Japan; 12 Department of Neurology Tohoku University Hospital Sendai Japan; 13 Data Science Juntendo University Graduate School of Medicine Tokyo Japan

**Keywords:** dry eye syndrome, dry eye disease, mobile health, smartphone, biobank, ocular surface, digital health, genome-wide association study

## Abstract

**Background:**

Dry eye disease (DED) is a common ocular condition with diverse and heterogeneous symptoms. Current treatment standards of DED include the post facto management of associated symptoms through topical eye drops. However, there is a need for predictive, preventive, personalized, and participatory medicine. The DryEyeRhythm mobile health app enables real-time data collection on environmental, lifestyle, host, and digital factors in a patient’s daily environment. Combining these data with genetic information from biobanks could enhance our understanding of individual variations and facilitate the development of personalized treatment strategies for DED.

**Objective:**

This study aims to integrate digital data from the DryEyeRhythm smartphone app with the Tohoku Medical Megabank database to create a comprehensive database that elucidates the interplay between multifactorial factors and the onset and progression of DED.

**Methods:**

This prospective observational cohort study will include 1200 participants for the discovery stage and 1000 participants for the replication stage, all of whom have data available in the Tohoku Medical Megabank database. Participants will be recruited from the Community Support Center of Sendai, Miyagi Prefecture, Japan. Participant enrollment for the discovery stage was conducted from August 1, 2021, to June 30, 2022, and the replication stage will be conducted from August 31, 2024, to March 31, 2026. Participants will provide demographic data, medical history, lifestyle information, DED symptoms, and maximum blink interval measurements at baseline and after 30 days using the DryEyeRhythm smartphone app. Upon scanning a registration code, each participant’s cohort ID from the Tohoku Medical Megabank database will be linked to their smartphone app, enabling data integration between the Tohoku Medical Megabank and DryEyeRhythm database. The primary outcome will assess the association between genetic polymorphisms and DED using a genome-wide association study. Secondary outcomes will explore associations between DED and various factors, including sociodemographic characteristics, lifestyle habits, medical history, biospecimen analyses (eg, blood and urine), and physiological measurements (eg, height, weight, and eye examination results). Associations will be evaluated using logistic regression analysis, adjusting for potential confounding factors.

**Results:**

The discovery stage of participant enrollment was conducted from August 1, 2021, to June 30, 2022. The replication stage will take place from August 31, 2024, to March 31, 2026. Data analysis is expected to be completed by September 2026, with results reported by March 2027.

**Conclusions:**

This study highlights the potential of smartphone apps in advancing biobank research and deepening the understanding of multifactorial DED, paving the way for personalized treatment strategies in the future.

**International Registered Report Identifier (IRRID):**

DERR1-10.2196/67862

## Introduction

Dry eye disease (DED) is one of the most diagnosed ocular conditions, with an estimated prevalence of 5%-50% [1]. It is associated with several recent global trends, including an aging population, a digitalized society, and cultural changes related to the COVID-19 pandemic [2-4]. The economic burden caused by the negative impact of DED on quality of life and vision is estimated at US $3.84 billion in the United States [5]. Patients with DED present with a highly heterogeneous range of symptoms, including dryness, photophobia, eye fatigue, and decreased visual acuity [6-8]. This situation presents a diagnostic challenge for clinicians, as several DED symptoms are nonspecific, resulting in a substantial number of patients being undiagnosed and untreated [9].

The progression of DED to severe stages, as observed in cases of Sjögren syndrome, graft-versus-host disease, and Stevens-Johnson syndrome, can lead to the development of corneal epithelial disorders that are refractory to existing therapies. Consequently, patients may experience severe pain, infectious keratitis, corneal perforations, and vision loss due to corneal neovascularization [10]. Current standards for DED treatment involve the post-facto management of associated symptoms using topical eye drops. However, no definitive cure exists for this chronic condition, which often affects individuals throughout their lives. Therefore, clinicians should actively implement preventive and predictive medicine strategies to prevent the onset and progression of DED. These strategies involve identifying risk factors and exploring new biomarkers to assist providers in anticipating disease progression [6-8].

From a pathophysiological perspective, DED is considered a multifactorial disease that disrupts tear film layer homeostasis [11]. Its onset and progression arise from the interplay of various factors, which are broadly divided into 3 categories: lifestyle factors (diet, cigarette use, physical activity, on-screen time, and contact lens use), environmental factors (humidity, pollen level, and particulate matter 2.5), and host factors (age, sex, presence of collagen diseases, family medical history, and genetic predispositions) [1]. Therefore, improving the current understanding and treatment of DED requires a comprehensive assessment of each patient’s health status. Several clinicians recommend effective strategies for preventive, predictive, personalized, and participatory medicine (P4 medicine). These strategies include a holistic review of associated factors using personalized digital data on lifestyle patterns and digital phenotypes obtained through mobile health (mHealth) apps and integrating mHealth data with comprehensive biomedical data, such as genomic composition and DED-related laboratory findings [1,12].

mHealth, as defined by the World Health Organization, refers to a broad range of “medical and public health practice supported by mobile devices, such as mobile phones, patient monitoring devices, personal digital assistants, and other wireless devices” [13]. Recent advancements in portable smart devices have accelerated global efforts to implement mHealth in clinical practice [14]. Compared with existing electronic medical record software or epidemiologic studies, mHealth-based studies facilitate the collection of personal longitudinal health data, such as daily symptoms and lifestyle factors, with minimal intrusion. They also provide access to different types of comprehensive digital data, including those from embedded biosensors and real-time environmental information available online [6-9,15]. mHealth enables the collection of longitudinal, high-frequency, real-time, and remote data for clinicians and researchers. In addition, it supports bidirectional participatory medicine, allowing users and participants to provide active feedback [16]. Informed consent for research participation can be obtained digitally, reducing access barriers and increasing outreach to traditionally secluded populations [17].

To facilitate the implementation of P4 medicine, we launched DryEyeRhythm, an in-house mHealth app for DED research, in 2016 and 2020 for iOS and Android, respectively. To date, DryEyeRhythm has enrolled over 55,000 participants who have provided comprehensive real-world data, including subjective symptoms, digital information, lifestyle factors, environmental status, and biosensor data [7,9,15,17-20]. For our digital cohort studies on DED variability and heterogeneity, the DryEyeRhythm app has been pivotal in monitoring digital data, lifestyle factors, environmental data, and symptom changes that reflect participants’ daily life and activity patterns.

A study based on the TwinsUK registry cohort [21] indicated that several chronic pain syndromes, including DED, demonstrated genetic heritability. A follow-up metabolome study revealed several metabolites associated with DED. Multiple gene loci are hypothesized to influence the onset and progression of DED, with several gene polymorphisms playing a crucial role in DED pathogenesis [22-39]. Polymorphisms in the estrogen receptor α and vitamin D receptor gene *APA-1* (rs7975232) have been associated with an increased prevalence of DED in several ethnic groups [22-24,28]. However, as of December 2023, no comprehensive reports on the genetic factors underlying DED heritability from genome-wide association studies (GWAS) have been published. Although numerous genetic factors contribute to DED heritability, the lack of GWAS limits the understanding of gene loci associated with DED onset and progression, hindering the provision of personalized medicine.

The Tohoku Medical Megabank (TMM) project was established in 2012 to support the reconstruction of areas affected by the Great East Japan Earthquake, promote the health of affected populations, and provide personalized and preventive medicine [40]. Collaborating with the Tohoku University and Iwate TMM Organizations in Miyagi and Iwate Prefectures, respectively, a 3-generation cohort study involving 150,000 local participants was conducted. The collected medical samples and data have established a foundation for a large-scale biobank [40-42].

This study conducts an add-on study to integrate DED-related personalized digital data, such as lifestyle factors and activity patterns, with the existing TMM databases by providing the DryEyeRhythm app to the participants of TMM cohort studies. These TMM databases include genomics, epidemiologic data, medical history, medical sample analyses, and physiological function testing results. Ultimately, we seek to establish a novel, comprehensive database for DED to elucidate the interplay between environmental factors, lifestyle choices, host factors, gene loci, and polymorphism traits that contribute to the onset and progression of DED, as well as to identify new DED subtypes through stratification techniques.

## Methods

### The TMM Project

As part of the TMM project, an integrated biobank was established based on the TMM Community-Based Cohort Study (TMM CommCohort Study) and TMM Birth and Three-Generation Cohort Study (TMM BirThree Cohort Study). The study design and recruitment methods have been described previously [40-42]. Briefly, the TMM CommCohort Study began in May 2013 and recruited 80,000 residents of the Miyagi and Iwate Prefecture in Japan. Participants responded to questionnaire items regarding sociodemographic factors, lifestyle habits, and medical history; provided biospecimens, such as blood and urine; and underwent physiological measurements, such as height, weight, and eye examinations [41-43]. The TMM BirThree Cohort Study commenced in July 2013 and recruited 70,000 residents (approximately 40,000 adults) of the Miyagi Prefecture. The survey items were nearly identical to those used in the TMM CommCohort Study [40-42]. The 2 cohort studies conducted follow-up surveys, including mailed questionnaires and repeated health examinations at community support centers in Miyagi Prefecture and satellites in Iwate Prefecture. Individual clinical and genomic data were incorporated into the TMM database (dbTMM) [42].

### DryEyeRhythm Smartphone App

**Figure 1:**
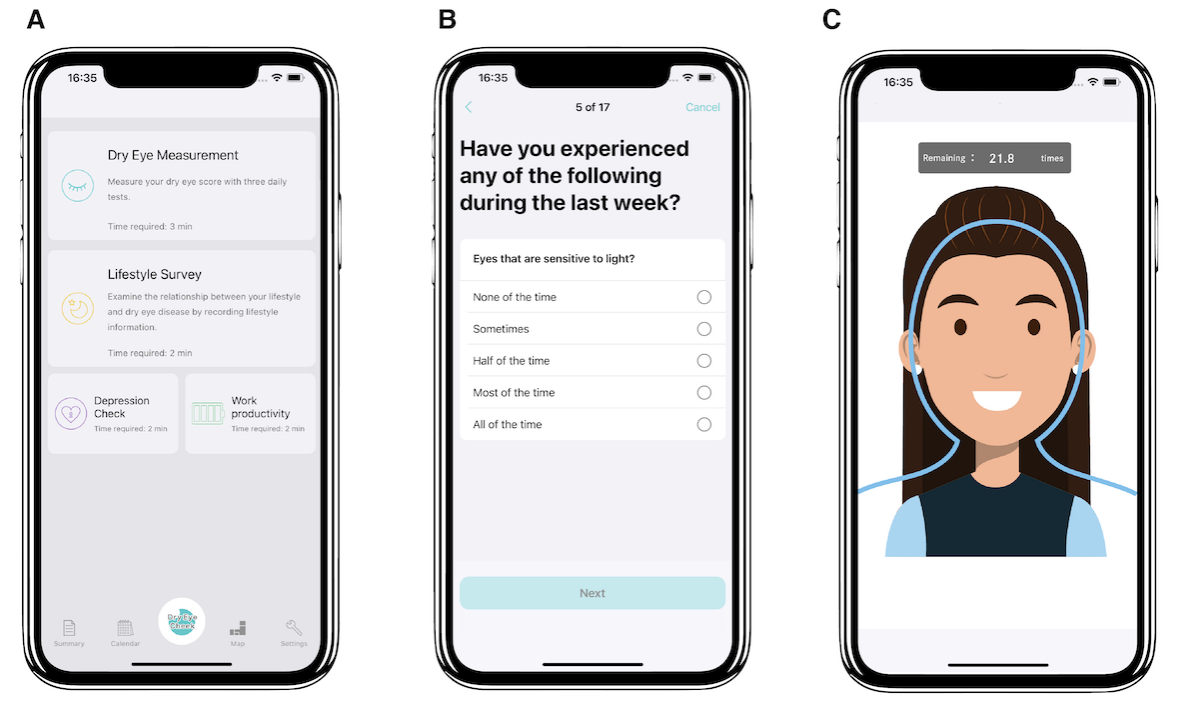
Screenshots of the DryEyeRhythm app. (A) Screenshot of the DryEyeRhythm test results. (B) screenshot of the DryEyeRhythm app–based J-OSDI. (C) Screenshot of the DryEyeRhythm exam menu. J-OSDI: Japanese version of the Ocular Surface Disease Index.

DryEyeRhythm, a free smartphone app for DED surveys and research, was launched in Japan in November 2016 and in the United States in April 2018 [15]. DryEyeRhythm collected electronic informed consent from all users. As illustrated in Figure 1A, participants provide demographic information, medical and lifestyle histories, and disease-specific questionnaire results for DED (Figure 1B, the Japanese version of the Ocular Surface Disease Index [J-OSDI]) [19,44], results of a depression rating scale results (Zung Self-rating Depression Scale [SDS]) [45], and work productivity details (Table S1 in Multimedia Appendix 1). Furthermore, DryEyeRhythm is equipped with functionality to measure the blink rate and maximum blink interval (MBI) of participants using smartphone cameras (Figure 1C) [7,20,46]. Collected data are automatically stored on the dedicated data server of the DryEyeRhythm smartphone app.

### Study Design and Participants

**Figure 2:**
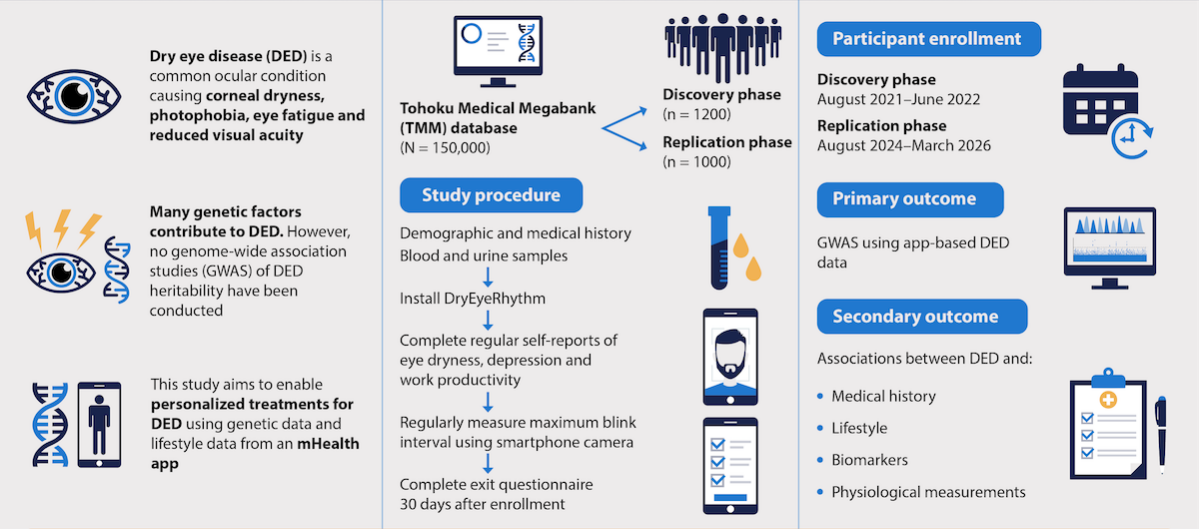
Schematic overview of the study design and participants.

This study was designed as a prospective observational add-on cohort study (Figure 2). Eligible participants were aged ≥20 years at the time of providing informed consent, participated in the TMM CommCohort Study or TMM BirThree Cohort Study, and conducted a follow-up survey at the Community Support Center of Sendai. A key inclusion criterion was that participants could use their smartphones. Participants were excluded if they did not have or use a smartphone or if their advice did not meet the operating system requirements (iOS 13.0 or later for iPhones; Android 8.0 or later for Android phones). Participants could withdraw their consent at any time before completing the second survey.

We aim to recruit participants at the Community Support Center of Sendai in Miyagi Prefecture, Japan, by distributing brochures when they present for health surveys. Those interested in the study have been and will be recruited. Study recruitment will occur between March 2021 and March 2026. Participants enrolled between March 2021 and June 2022 were included in the discovery stage cohort, whereas those enrolled between August 2024 and March 2026 will be included in the replication stage cohort. Participants will download and activate the DryEyeRhythm app [15] when they are recruited, and the cohort ID assigned to each participant will be scanned using a smartphone camera to connect the cohort ID to the app. The participants will be required to review the study description and provide electronic informed consent by DryEyeRhythm. The DryEyeRhythm survey can be completed anywhere; however, participants who complete the survey at the Community Support Center of Sendai can receive assistance from genome medical research coordinators if they have questions regarding the survey.

### Study Procedures

**Figure 3:**
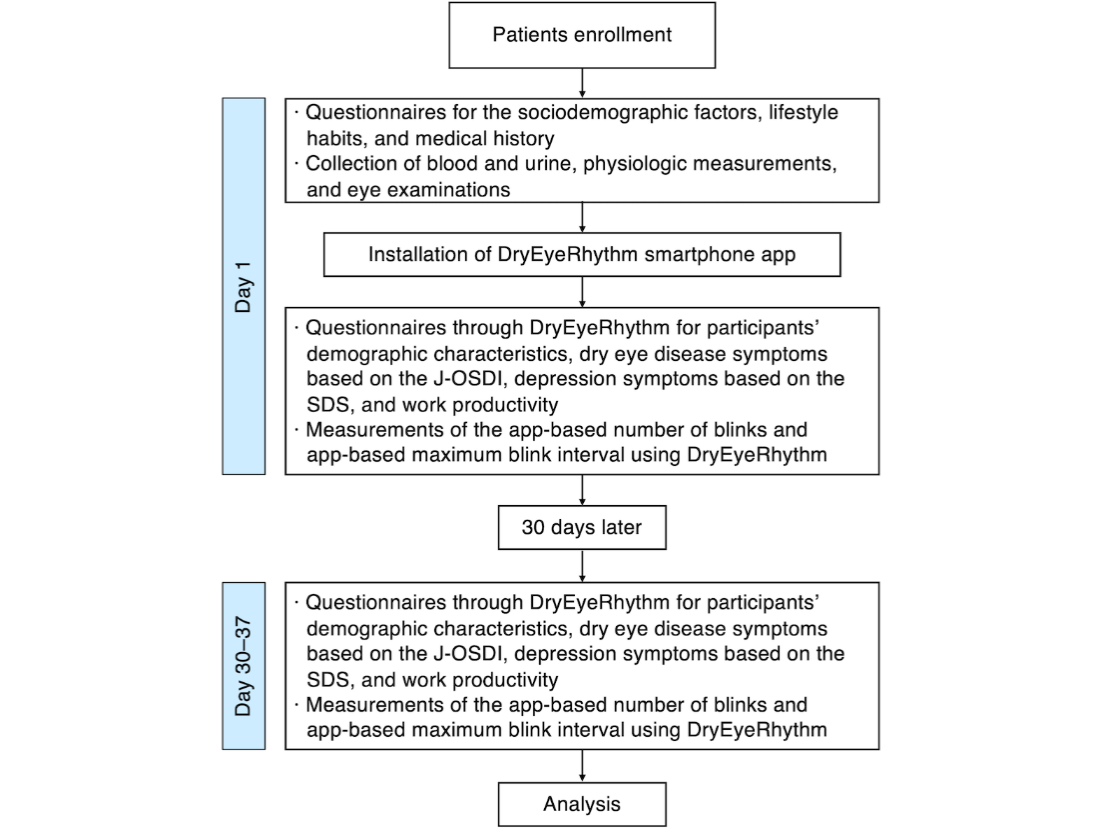
Flowchart of this study. J-OSDI: Japanese version of the Ocular Surface Disease Index; SDS: Zung Self-rating Depression Scale.

Figure 3 shows a flowchart of the study. Participants visiting the Community Support Center of Sendai for health surveys and providing consent will complete questionnaires on sociodemographic factors, lifestyle habits, and medical history. They will also undergo blood and urine collection, physiological measurements, and eye examinations. Subsequently, participants will install the DryEyeRhythm app on their smartphones. They will then provide data through DryEyeRhythm (Figure 1A) regarding their demographic characteristics, medical and lifestyle histories, DED symptoms based on the J-OSDI (Figure 1B), depression symptoms based on the SDS, work productivity, and app-based blink rate and MBI (Figure 1C) measurements through the smartphone camera. After 30 days, participants who receive a notification requesting a second round of data submission will provide another round of information on DED symptoms based on the same criteria as the first round using DryEyeRhythm. Participants who do not submit the second-round data will receive a reminder through a phone call.

### DED Diagnosis

Subjective symptoms of DED will be assessed using the 12-item J-OSDI questionnaire with 3 subscales: ocular symptoms, visual functioning, and environmental triggers [47]. Each response will be recorded on a 5-point scale (0=“None of the time” to 4=“All of the time” [4]), with “N/A” for questions not applicable to the user. The J-OSDI total score will be reported on a 100-point scale to determine the severity of DED symptoms (0-12, normal; 13-22, mild; 23-33, moderate; and 33-100, severe). The app-based J-OSDI has been validated in Japanese [44]. In this study, subjective symptoms of DED will be evaluated using the DryEyeRhythm-based (app-based) J-OSDI (Figure 1B). The app-based J-OSDI has been validated and compared with the paper-based J-OSDI in a previous study [19].

The MBI is defined as the duration the participants could keep their eyes open [46]. MBI is positively correlated with tear film breakup time (TFBUT) [48]. In this study, the app-based MBI measured using DryEyeRhythm (Figure 1C) will be used for diagnosing DED. A previous study has demonstrated that the app-based MBI is a valid and reliable substitute measurement compared with the slit-lamp-based MBI [20].

We defined app-based DED as an app-based J-OSDI total score of ≥ 13 points and app-based MBI of ≤ 12.4 seconds [48]. The app-based diagnostic method has been validated as a simple and noninvasive screening test for DED in previous studies [17,48]. The sensitivity, specificity, and area under the curve values for the app-based diagnostic method were 50%, 95%, and 0.91, respectively.

### GWAS for the Primary Outcome

Genotyping of participants and data imputations will be completed using TMM, as previously described [49]. Individuals will be excluded from the analysis if they have a low call rate (<0.95), deviate from the mean of the major population by more than 4 SDs in genotyping principal component (PC) 1 and PC2, a medical history of Parkinson disease, or lack phenotypic and covariate data such as age, sex, and PCs. Variants will be excluded if they have a low call rate (<0.99), low Hardy-Weinberg equilibrium exact test *P* value (*P*<1×10^–6^), low minor allele frequency (<0.01), or low imputation quality (*R*^2^<0.3). The target outcome will be app-based DED. A stringent threshold of *P*<5×10^–8^ will denote genome-wide significance, minimizing false positives associated with multiple testing, and a suggestive threshold of *P*<1×10^–5^ to indicate potential associations that warrant further investigation. Only variants reaching the threshold (*P*<5×10⁻^5^) during the discovery stage will be selected for the replication stage. Logistic regression will be applied to binary outcomes, adjusting for confounding factors (such as age, sex, and PCs) using well-validated GWAS tools, including Plink2 [50].

### Secondary Outcome

The secondary outcomes of this study will include the association between app-based DED and patients’ sociodemographic factors, lifestyle habits, medical history, biospecimens such as blood and urine, and physiological measurements such as height, weight, and eye examination results [41-43]. These associations will be evaluated using logistic regression analysis and adjusted for confounding factors. A significance level of *P*<.05 will be used.

### Sample Size Calculation

This study’s sample size was calculated using the web-based tool Genetic Association Study Power Calculator [51]. The rs1143634 polymorphism in IL1B was selected as a candidate single-nucleotide polymorphism based on previous associations with non-Sjögren DED, with an odds ratio of 3.337 [26]. The minor allele frequency for rs1143634 was 0.047, based on data from the Japanese Multi Omics Reference Panel [52]. The prevalence of DED in the target population was estimated to be 30% [53], with a significance level of *P*<5×10⁻⁸. The sample size was calculated to achieve an 80% statistical power, assuming 2000 participants (600 cases and 1400 controls, with a 30:70 case-control ratio).

**Figure 4:**
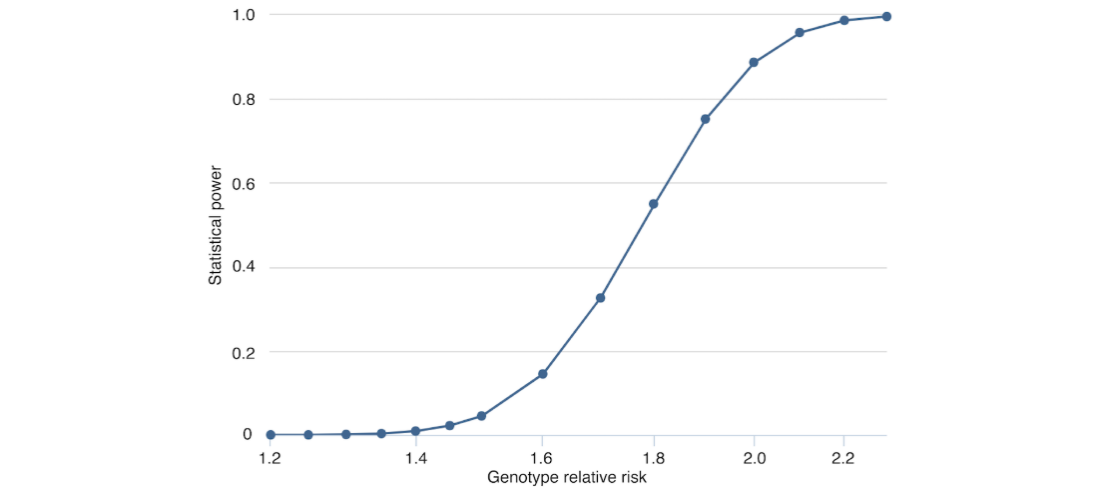
Statistical power analysis.

As shown in Figure 4, the statistical power increased as the relative risk approached ≥1.9. The odds ratio can be used as an approximation of the relative risk. With an odds ratio of 3.337 for rs1143634, the sample size was deemed adequate for detecting associations based on the effect size observed in a previous study [26]. The sample size was increased by 10% to account for potential exclusions, resulting in a final recruitment target of 2200 participants. Of these, 1200 participants were included in the discovery stage, and 1000 participants will be included in the replication stage.

### Statistical Analyses

The initial data selection for the DryEyeRhythm dataset collected will be conducted for each cohort ID. If the dataset contains complete data (data without missing values), the first available dataset will be used as the initial dataset. If only incomplete data are registered, the first incomplete dataset will be used as the initial dataset. If complete data are registered on or after 30 days and before 37 days from the initial dataset, the first dataset among them will be used as the second dataset. If only incomplete data are registered within this period, the first dataset among them will be used as the second dataset. The demographic characteristics of the participants will be analyzed as the input values of the initial data. The month of a participant’s inclusion will be defined as the month when their initial data are recorded. The ophthalmic data analyzed in this study will be measured at the time of recruitment for the TMM CommCohort Study and TMM BirThree Cohort Study and stored in the dbTMM [40-42].

Continuous variables will be presented as median (IQR) values and categorical variables as percentages. The Mann-Whitney *U* test will be used for continuous variables, and the chi-square test for categorical variables. All analyses will be conducted with a significance level of .05 using a 2-sided test. The associations between app-based DED and factors stored in the dbTMM and measured through DryEyeRhythm will be evaluated using the logistic regression analysis. This model will incorporate participants’ characteristics (such as age, gender, height, weight, and medical history), lifestyle factors, environmental factors (including residential environment), and ophthalmic examination results as variables to calculate fully adjusted odds ratios, accounting for various confounding factors. All data will be analyzed using Stata (version 18.0; StataCorp) and R (version 4.2.1; R Foundation for Statistical Computing).

### Ethical Considerations

This study was approved by the Ethics Committee of the Tohoku University TMM Organization (approval number: 2023-4-188; September 10, 2023, version 1), the Independent Ethics Committee of Juntendo University Faculty of Medicine, and the Ethics Committee of Iwate Medical University, adhering to the principles outlined in the tenets of the Declaration of Helsinki. The research protocol was reviewed by the Ethics Committee of the Tohoku University TMM Organization on behalf of multiple institutions. All participants will provide informed consent upon visiting the Community Support Center, Sendai, Miyagi, Japan. In addition, electronic informed consent will be obtained upon the first activation of the DryEyeRhythm smartphone app. The consent process includes approval for the use of data from the Tohoku Medical Megabank database. To ensure participants’ confidentiality, all data will be anonymized, with participants identified using a cohort ID that prevents personal identification. Participants will receive an honorarium of 500 Yen (US $3.50) per survey (up to a maximum of 1000 Yen [US $7]) for completing the DryEyeRhythm surveys.

## Results

Participant enrollment for the discovery stage was performed from August 1, 2021, to June 30, 2022. Enrollment for the replication stage will be performed from August 31, 2024, to March 31, 2026. Data analysis will be completed by September 2026, and results will be reported by March 2027.

## Discussion

Implementing the principles of P4 medicine through cross-hierarchical analyses, which include comprehensive and personalized digital health and genomic datasets, may be crucial for preventing the onset or progression of highly multifactorial DED. By integrating DryEyeRhythm, our mHealth app for DED research, with the TMM project, we establish a comprehensive database for DED that integrates genomic data from the biobank with multifaceted datasets (ie, lifestyle, biosensor records, and demographics) collected through our app. Using this robust dataset, we identify new gene loci and polymorphisms associated with DED pathogenesis and progression, as well as new DED subtypes and their characteristics through stratification strategies. This approach can also be extended to other fields of medicine, providing insights into the underlying pathophysiology of various multifactorial diseases.

We conducted a systematic review using PubMed and Embase for all research articles published until April 15, 2024, using the search terms “(dry eye) AND ((genome) OR (polymorphism) OR (SNP) OR (variant) OR (locus) OR (loci) (mutation)).” This systematic review did not identify any existing publications that collected real-world, daily DED-related health data, such as lifestyle patterns, environmental status, biosensor inputs, or subjective symptoms using mHealth technology. However, we identified 20 reports on gene mutations or polymorphisms associated with DED onset and severity [22-39,54,55]. No studies have conducted GWAS to identify loci associated with DED. Therefore, the proposed approach marks the first attempt to create a holistic database for DED using genomic and digital health data. This integration may reveal unknown aspects of DED regarding its variability, heterogeneity, and implementation of P4 medicine. In addition, we highlight the new use and value of biobanks in global health care research communities through this initiative, eventually promoting the incorporation of genomic studies into standard clinical practice.

The add-on portion of this study will be conducted through DryEyeRhythm to provide DED diagnostic support [15]. The diagnostic criteria for DED in Japan include positive subjective symptoms on the DED-specific symptom questionnaire and decreased TFBUT [44,56]. To meet these criteria, DryEyeRhythm first administers the J-OSDI questionnaire through its app interface [17,46,48]. Instead of directly measuring TFBUT, the app measures the MBI of the participants, which is positively correlated with TFBUT [17,46,48]. Our previous studies on the comeasurement of the app-based J-OSDI total score and MBI demonstrated sufficient validity, reliability, and equivalence to the traditional diagnostic standard [19,20]. Regarding diagnostic performance, the positive and negative predictive values were reported to be 91.3% and 69.1%, respectively [17]. Furthermore, using data collected through DryEyeRhythm, we developed and tested an original stratification algorithm that identified 7 DED subtypes based on subjective symptoms [7]. In this study, we applied the above stratification technique to better detect DED subtypes and understand DED pathophysiology by identifying the digital phenotypic characteristics of each cluster.

Another crucial aspect of this protocol is the cross-hierarchical analysis using real-time, day-to-day digital data and lifestyle factors, which are difficult to capture in traditional cohort studies involving established biobanks [6,7,9,15]. Typically, biobanks require participants to visit facilities or send samples directly for collection. Conversely, mHealth offers the advantage of nonintrusive, longitudinal data collection, closely reflecting real-world data as facility-attained data are readily affected by undesired variables (such as white-coat syndrome and masked hypertension) [57]. Such data characteristics that closely resemble real-world data can be particularly crucial for perfecting the future implementation of P4 medicine [57]. For DED, day-to-day health data can be important as they are associated with daily activities and factors immeasurable in a facility setting, such as on-screen time and living environment. With the use of common smart devices and attached sensors, clinicians and researchers can access the data above. Objective data points, including DED questionnaire scores and biosensor data, can reduce barriers to access and cost, as mHealth apps can be operated by users without requiring specialized examination tools or personnel [17,19,20,58]. Physical and biological specimens such as genomic samples, secretions, or hair will still be crucial but with a shift toward using mailed specimens, allowing for a study cohort without geographic limitations to populations near specific research centers. Cohort studies in Western countries where participants provided consent through a smartphone app and physical specimens through the mail have been reported [59,60]. Although no similar attempts have been made in ophthalmology, DryEyeRhythm could serve as a platform to recruit cohorts similarly and expand the use of existing and prospective biobanks.

Our protocol has some limitations, consistent with those identified in previous studies using smartphone apps [9,15]. First, this study may be prone to recall bias due to the reliance on self-administered questionnaires, which may lead to an overestimation of DED prevalence in the community. On the other hand, in evaluating subjective symptoms of DED, self-reported questionnaires, such as the J-OSDI questionnaire, are commonly used in standard clinical DED diagnostic methods [56]. Therefore, the impact of using a self-reported questionnaire for subjective DED symptoms on the study results is likely minimal. Second, selection bias may occur, as individuals concerned about DED prevalence and those with previous symptoms may be more likely to participate due to the voluntary registration process. In addition, older adults or those lacking access to smart devices will have difficulty participating in this study. This study will target participants of the TMM CommCohort Study and the TMM BirThree Cohort Study, which geographically restricts the pool of participants. The limitations in the geographical and demographic representativeness of the sample may introduce bias and affect the generalizability of the study results. Third, this study will diagnose DED using app-based J-OSDI and MBI metrics without conducting clinical examinations, such as TFBUT, the Schirmer test, osmolality measurements, and ocular surface staining [9,11,46]. The difference in this method may affect the study results. However, this app-based DED diagnostic method has been validated for reliability and validity against the standard DED diagnostic method, which uses the paper-based J-OSDI questionnaire and TFBUT [17,56]. The positive predictive and negative predictive values of the app-based DED assessment were 91.3% and 69.1%, respectively, with an area under the curve of 0.91. Therefore, the effect of differences in the method of diagnosis of DED on the results of this study is likely minimal.

In conclusion, by establishing a comprehensive database for DED that integrates existing medical biobank data with highly personalized and holistic mHealth data, this study may discover a new biobank value and contribute to the implementation of genomics in clinical practice.

## Appendix

Multimedia Appendix 1 DryEyeRhythm questions.

## Abbreviations

dbTMM: database Tohoku Medical Megabank
DED: dry eye disease
GWAS: genome-wide association studies
J-OSDI: Japanese version of the Ocular Surface Disease Index
MBI: maximum blink interval
mHealth: mobile health
PC: principal component
P4 medicine: predictive, preventive, personalized, and participatory medicine
SDS: Zung Self-rating Depression Scale
TFBUT: tear film breakup time
TMM: Tohoku Medical Megabank
TMM BirThree Cohort: TMM Birth and Three-Generation Cohort Study
TMM CommCohort: TMM Community-Based Cohort Study

## Data Availability

All data are available in the main text. The code supporting the findings of this study will be made available from the corresponding author upon reasonable request. Data processing and analysis will be performed using STATA version 18.0.
