# Supplementary material for: Integration of Digital Phenotyping and Genomics for Dry Eye Disease: Protocol for a Prospective Cohort Study
Source: JMIR Res Protoc. 2025 May 12;14:e67862. doi: 10.2196/67862 (PMC12107206; doi:10.2196/67862)
Supplement: Multimedia Appendix 1 [file resprot_v14i1e67862_app1.pdf]

Supplemental Table 1. DryEyeRhythm questions.

Class	Question	Variables	Variable details
Basic information			
		Height	Integer input, cm
		Weight	Integer input, kg
		Age	Integer input, years
		Sex	Choose one {"Men," "Women"}
		Race	Choose one {"American Indian or Alaskan Native," "Arabic," "Asian," "Black or African American," "Caucasian," "Hispanic, Latino, or Spanish," "Native Hawaiian or other Pacific Islander," "Other Race"}
	Are you currently enrolled in a school？	Education	Choose one {“Student (currently enrolled)”, “Graduated”}
	What is your highest level of education attained? If you are a current student, tell us your current affiliation	Final education	Choose one {“Elementary・Middle School”, “High School”, “Professional School”, “Junior School・Technical School”, “Undergraduate School”, ”Graduate School”}
	What is your personal (not household) annual income in units of 10k yen?	Salary	Choose one {“0-$199k”, “$200k-$399k”, “$400k-$599k”, “$600k-$799k”, “$800k-$999k”, “$1,000k-$1,499k”, “$1,500k-$1,999k”, “above $2,000k”}

	If you do not have income, please select less than $199k Can you exchange dollars? Rate is 1dollar = 100 yen.		
	Please select all medicine you regularly take from the following list:	Medicine	Multiple choice among {"Blood pressure medication (Antihypertensive)", "Diabetes medication", "Antihyperlipidemic agents (i.e. cholesterol- and lipid-lowering drugs)", "Diuretics (water pills)", "Antihistamines (allergy drugs", "Antidepressants", "Anxiolytics (anti-anxiety/anti-panic drugs)", "Hormone replacement therapy", "Oral contraceptive pills (birth control pills)", "Multi-vitamin supplements", "None"}
	Have you ever had any of the following diseases in the past?	Medical history	Multiple choice among {"Cardiac (Heart-related)", "Respiratory (Lung-related)", "Neurologic (Nerve- or Brain-related)", "Hepatic (Liver-related)", "Renal (Kidney-related)", "Thyroid", "Hematologic (Blood-related)", "Malignancy (Cancer-related)", "None above"}
	Do you have any ongoing mental illness(es) from the following list?	Mental illness	Multiple choice among {"Major Depressive Disorder (Depression)", "Anxiety-related Disorder (including generalized anxiety, phobias, and PTSD)", "Schizophrenia", "Bipolar Disorder (Manic Depression)", "Other psychologic disorders", "None"}
	Have you ever been diagnosed with dry eye by an ophthalmologist?	Past diagnosis of dry eye disease	Choose one {"Yes", "No"}

	Enter the right eyesight (the naked eye)<br>Please round the number to the closest 1 decimal point. (0.1)	Right visual acuity	Choose one {“less than 20/200”, “20//200”, “20/160”, “20/120”, “20/100”, “20/80”, “20/60”, “20/50”, “20/40”, “20/30”, “20/25”, “20/20”, “20/15”}
	Enter the left eyesight (the naked eye)<br>Please round the number to the closest 1 decimal point. (0.1)	Left visual acuity	Choose one {“less than 20/200”, “20//200”, “20/160”, “20/120”, “20/100”, “20/80”, “20/60”, “20/50”, “20/40”, “20/30”, “20/25”, “20/20”, “20/15”}
	Have you ever used contact lenses?	Contact lens use	Choose one {“I have been using contact lenses”, “I used contact lenses in the past”, “I have not used contact lenses”}
	Which of the following types of contact lens do you use? (Or did you use?)	Contact lens type	Multiple choice among {“Daily disposable contact lenses”, “2-week replaceable contact lenses”, “1-month replaceable contact lenses”, “Regular long-term contact lenses (Soft contact lens)”, “Regular long-term contact lenses (Hard contact lens)”, “Orthokeratology (Ortho-K, overnight vision correction contact lenses)”
	Which of the following types of contact lenses do you use? (Or did you use?)	Contact lens material	Multiple choice among {“Soft contact lenses”, “Silicone contact lenses”, “Color contact lenses”, “Hard contact lenses”, “Orthokeratology (Ortho-K, overnight vision correction contact lenses)”}

	How long have you been using contact lenses?	Contact lens term	Integer input, years
	How long do you wear your contact lenses per day?	Contact lens duration per day	Integer input (0–24), hours
	Do you use eye drops for dry eyes?	Eye drop	Choose one {“Yes,” “No”}
	What kind of eye drops do you use?	Eye drop	Multiple choice among {“Prescription eye drops”, “Over-the-counter eye drops”}
	Have you had any of the following eye surgeries?	Ophthalmic surgery	Multiple choice among {“Cataract”, “Refractive Surgery (i.e. LASIK, PRK, LASEK, etc.)”, “Other Ophthalmic Surgery”, “None”}
	How many hours do you exercise per week?	Periodic exercise	Integer input, hours
	Do you (or have you) smoke(d) cigarettes, including heat-not-smoke cigarettes?	Smoking	Choose one {“Active Smoker”, “Past Smoker”, “Never Smoked”}
	How many cigarettes do you (or have you) smoke(d) per day?	Smoking amount	Integer input, cigarettes

	How long did you (or have you) smoke(d)?	Smoking term	Integer input, years
	Do you have hay fever (allergic rhinitis)?	Hay fever	Choose one {“Yes”, “No”}
Dry eye measurement			
	Count the number of blinks.	Number of blinks	Measure number of blinks.
	Measure how many seconds you can tolerate without blinking.	Maximum interval between blinks	Measure maximum interval between blinks.
	Have you experienced any of the following during the last week?<br>Eyes that are sensitive to light?	Dazzling (J-OSDI 1)	Choose one {“None of the time”, “Sometimes”, “Half of the time”, “Most of the time”, “All of the time”}
	Have you experienced any of the following during the last week?<br>Eyes that feel gritty/sandy?	Discomfort (J-OSDI 2)	Choose one {“None of the time”, “Sometimes”, “Half of the time”, “Most of the time”, “All of the time”}

	Have you experienced any of the following during the last week?<br>Painful or sore eyes?	Painful<br>(J-OSDI 3)	Choose one {“None of the time”, “Sometimes”, “Half of the time”, “Most of the time”, “All of the time”}
	Have you experienced any of the following during the last week?<br>Blurry vision?	Grow hazy<br>(J-OSDI 4)	Choose one {“None of the time”, “Sometimes”, “Half of the time”, “Most of the time”, “All of the time”}
	Have you experienced any of the following during the last week?<br>Poor vision?	Hard to see<br>(J-OSDI 5)	Choose one {“None of the time”, “Sometimes”, “Half of the time”, “Most of the time”, “All of the time”}
	Have you experienced any of the following during the last week?<br>Reading?	Reading<br>(J-OSDI 6)	Choose one {“None of the time”, “Sometimes”, “Half of the time”, “Most of the time”, “All of the time”, “N/A”}
	Have you experienced any of the following during the last week?<br>Driving at night?	Night driving (J-OSDI 7)	Choose one {“None of the time”, “Sometimes”, “Half of the time”, “Most of the time”, “All of the time”, “N/A”}
	Have you experienced any of the following during the last week?	PC<br>(J-OSDI 8)	Choose one {“None of the time”, “Sometimes”, “Half of the time”, “Most of the time”, “All of the time”, “N/A”}

	Working with a computer or bank machine (ATM)?		
	Have you experienced any of the following during the last week?<br>Watching TV?	Watch TV (J-OSDI 9)	Choose one {"None of the time", "Sometimes", "Half of the time", "Most of the time", "All of the time", "N/A"}
	Have you experienced any of the following during the last week?<br>Windy conditions?	Wind (J-OSDI 10)	Choose one {"None of the time", "Sometimes", "Half of the time", "Most of the time", "All of the time", "N/A"}
	Have you experienced any of the following during the last week?<br>Places or areas with low humidity (very dry)?	Dry (J-OSDI 11)	Choose one {"None of the time", "Sometimes", "Half of the time", "Most of the time", "All of the time", "N/A"}
	Have you experienced any of the following during the last week<br>Areas that are air conditioned?	Air conditioner (J-OSDI 12)	Choose one {"None of the time", "Sometimes", "Half of the time", "Most of the time", "All of the time", "N/A"}
Lifestyle habit			

	Please answer each question using a 5point scale	Stress level	Scale bar input (0 to 5; 0 = No stress at all, 5 = Feels intense stress)
	Have you had any of the following symptoms in the last week?<br>Headache	Headache	Choose one {“None of the time”, “Sometimes”, “Half of the time”, “Most of the time”, “All the time”, “N/A″}
	Have you had any of the following symptoms in the last week?<br>Eye itching	Itching	Choose one {“None of the time”, “Sometimes”, “Half of the time”, “Most of the time”, “All the time”, “N/A″}
	Have you had any of the following symptoms in the last week?<br>Asthenopia (eye strain)	Eye strain	Choose one {“None of the time”, “Sometimes”, “Half of the time”, “Most of the time”, “All the time”, “N/A″}
	Have you had any of the following symptoms in the last week?<br>Muscle Stiffness/Pain	Stiffness	Choose one {“None of the time”, “Sometimes”, “Half of the time”, “Most of the time”, “All the time”, “N/A″}
	Have you had any of the following symptoms in the last week?<br>Mental or Emotional fatigue	Neuro psychiatric fatigue	Choose one {“None of the time”, “Sometimes”, “Half of the time”, “Most of the time”, “All the time”, “N/A″}

	Please tell me your bedtime last night.	Bedtime	Drum bar input, hours and minutes
	Please select your typical sleeping hours.	Sleeping time	Scale bar input (0–24), hours
	Please select your total step count from yesterday.<br>Please select the closest approximation of your step count.	Pedometer daily	Choose one {“0–999 steps”, “1,000–1,999 steps”, “2,000–2,999 steps”, “3,000–3,999 steps”, “4,000–4,999 steps”, “5,000–5,999 steps”, “6,000–6,999 steps”, “7,000–7,999 steps”, “8,000–8,999 steps”, “9,000–9,999 steps”, “10,000–10,999 steps”, “11,000–11,999 steps”, “12,000–12,999 steps”, “13,000–13,999 steps”, “14,000–14,999 steps”, “more than 15,000 steps”}
	Please tell me how much screen time exposure you had today.<br>Smartphone, PC, TV, etc.	Monitoring time	Scale bar input (0–24), hours
	How much coffee did you drink today?<br>One glass = approximately 180ml	Coffee intake	Scale bar input (0–10), glasses
	How much fluid did you drink today?	Water intake	Scale bar input (0–5,000), mL
Work productivity			

	Are you currently working (for a compensation, such as income).	Job status	Choose one {"Yes", "No"}
	How many hours did you work (realistically) in the past week?	Work hours	Scale bar input (0–168), hours
	How many hours did you rest during working hours in the past week due to dry eyes? Please include any time taken off, including absences, tardiness, and early leaves, due to health problems. Note: Time taken off to participate in this trial should not be included.	Dry eye effect – rest time of work	Scale bar input (0–168), hours
	How much did dry eye affect your productivity (any type and number of activities that could have been done) in the past week?	Dry eye effect – Work productivity	Scale bar input (0–10)
	Are you currently enrolled in a school as a student?	Student status	Choose one {"Yes", "No"}

	How many hours of class have you taken in the past week?	Class hours	Scale bar input (0–168), hours
	How many hours of class did you miss in the past week due to dry eyes?<br>Please include any time taken off, including absences, tardiness, and early leaves, due to health problems.<br>Note: Time taken off to participate in this trial should not be included.	Dry eye effect – missing a class	Scale bar input (0–168), hours
	How much did dry eye affect your studies in the past week?	Dry eye effect – study	Scale bar input (0–10)
	How much did dry eye affect your extra-curricular (not related to school or work) activities in the past week?	Dry eye effect – extra-curricular activities	Scale bar input (0–10)
Depression check			

	I feel down and blue	Depressed affect (SDS 1)	Scale bar input (1-4; 1 = Rarely, 2 = Some (1-2days), 3 = Occasionally (3-4days), 4 = Most)
	I feel best in the morning	Diurnal variation (SDS 2)	Scale bar input (1-4; 1 = Rarely, 2 = Some (1-2days), 3 = Occasionally (3-4days), 4 = Most)
	I have crying spells or feel like crying	Crying spells (SDS 3)	Scale bar input (1-4; 1 = Rarely, 2 = Some (1-2days), 3 = Occasionally (3-4days), 4 = Most)
	I have trouble sleeping at night	Sleep disturbance (SDS 4)	Scale bar input (1-4; 1 = Rarely, 2 = Some (1-2days), 3 = Occasionally (3-4days), 4 = Most)
	I eat as much as I used to	Decreased appetite (SDS 5)	Scale bar input (1-4; 1 = Rarely, 2 = Some (1-2days), 3 = Occasionally (3-4days), 4 = Most)
	I still enjoy sex, or am interested in seeking for a partner	Decreased libido (SDS 6)	Scale bar input (1-4; 1 = Rarely, 2 = Some (1-2days), 3 = Occasionally (3-4days), 4 = Most)
	I notice that I am losing weight	Weight loss (SDS 7)	Scale bar input (1-4; 1 = Rarely, 2 = Some (1-2days), 3 = Occasionally (3-4days), 4 = Most)
	I experience constipation	Constipation (SDS 8)	Scale bar input (1-4; 1 = Rarely, 2 = Some (1-2days), 3 = Occasionally (3-4days), 4 = Most)
	My heart beats faster than usual	Tachycardia (SDS9)	Scale bar input (1-4; 1 = Rarely, 2 = Some (1-2days), 3 = Occasionally (3-4days), 4 = Most)
	I get tired for no reason	Fatigue (SDS 10)	Scale bar input (1-4; 1 = Rarely, 2 = Some (1-2days), 3 = Occasionally (3-4days), 4 = Most)
	My mind is as clear as it used to be	Confusion (SDS 11)	Scale bar input (1-4; 1 = Rarely, 2 = Some (1-2days), 3 = Occasionally (3-4days), 4 = Most)

	I find it easy to do the things I used to do	Psychomotor retardation (SDS 12)	Scale bar input (1-4; 1 = Rarely, 2 = Some (1-2days), 3 = Occasionally (3-4days), 4 = Most)
	I am restless and can't keep still	Psychomotor agitation (SDS 13)	Scale bar input (1-4; 1 = Rarely, 2 = Some (1-2days), 3 = Occasionally (3-4days), 4 = Most)
	I feel there is hope in the future	Hopelessness (SDS 14)	Scale bar input (1-4; 1 = Rarely, 2 = Some (1-2days), 3 = Occasionally (3-4days), 4 = Most)
	I am more irritable than usual	Irritability (SDS 15)	Scale bar input (1-4; 1 = Rarely, 2 = Some (1-2days), 3 = Occasionally (3-4days), 4 = Most)
	I find it easy to make decisions	Indecisiveness (SDS 16)	Scale bar input (1-4; 1 = Rarely, 2 = Some (1-2days), 3 = Occasionally (3-4days), 4 = Most)
	I feel that I am useful and needed	Personal devaluation (SDS 17)	Scale bar input (1-4; 1 = Rarely, 2 = Some (1-2days), 3 = Occasionally (3-4days), 4 = Most)
	My life is pretty fulfilling	Emptiness (SDS 18)	Scale bar input (1-4; 1 = Rarely, 2 = Some (1-2days), 3 = Occasionally (3-4days), 4 = Most)
	I feel that others would be better off if I were dead	Suicidal rumination (SDS 19)	Scale bar input (1-4; 1 = Rarely, 2 = Some (1-2days), 3 = Occasionally (3-4days), 4 = Most)
	I still enjoy the things I used to do	Dissatisfaction (SDS 20)	Scale bar input (1-4; 1 = Rarely, 2 = Some (1-2days), 3 = Occasionally (3-4days), 4 = Most)

Abbreviations: LASIK: laser-assisted *in situ* keratomileusis; N/A: not applicable; PC: personal computer; TV: television.
